# Analyzing musculoskeletal neck pain, measured as present pain and periods of pain, with three different regression models: a cohort study

**DOI:** 10.1186/1471-2474-10-73

**Published:** 2009-06-19

**Authors:** Anna Grimby-Ekman, Eva M Andersson, Mats Hagberg

**Affiliations:** 1 Sahlgrenska School of Public Health and Community Medicine, University of Gothenburg (UGOT), Gothenburg, Sweden

## Abstract

**Background:**

In the literature there are discussions on the choice of outcome and the need for more longitudinal studies of musculoskeletal disorders. The general aim of this longitudinal study was to analyze musculoskeletal neck pain, in a group of young adults. Specific aims were to determine whether psychosocial factors, computer use, high work/study demands, and lifestyle are long-term or short-term factors for musculoskeletal neck pain, and whether these factors are important for developing or ongoing musculoskeletal neck pain.

**Methods:**

Three regression models were used to analyze the different outcomes. Pain at present was analyzed with a marginal logistic model, for number of years with pain a Poisson regression model was used and for developing and ongoing pain a logistic model was used. Presented results are odds ratios and proportion ratios (logistic models) and rate ratios (Poisson model). The material consisted of web-based questionnaires answered by 1204 Swedish university students from a prospective cohort recruited in 2002.

**Results:**

Perceived stress was a risk factor for pain at present (PR = 1.6), for developing pain (PR = 1.7) and for number of years with pain (RR = 1.3). High work/study demands was associated with pain at present (PR = 1.6); and with number of years with pain when the demands negatively affect home life (RR = 1.3). Computer use pattern (number of times/week with a computer session ≥ 4 h, without break) was a risk factor for developing pain (PR = 1.7), but also associated with pain at present (PR = 1.4) and number of years with pain (RR = 1.2). Among life style factors smoking (PR = 1.8) was found to be associated to pain at present. The difference between men and women in prevalence of musculoskeletal pain was confirmed in this study. It was smallest for the outcome ongoing pain (PR = 1.4) compared to pain at present (PR = 2.4) and developing pain (PR = 2.5).

**Conclusion:**

By using different regression models different aspects of neck pain pattern could be addressed and the risk factors impact on pain pattern was identified. Short-term risk factors were perceived stress, high work/study demands and computer use pattern (break pattern). Those were also long-term risk factors. For developing pain perceived stress and computer use pattern were risk factors.

## Background

The literature on musculoskeletal disorders contains ongoing discussions concerning both the relevance of the measured outcomes [1-4] and the lack of longitudinal studies [5,6]. Many previous studies of upper body musculoskeletal disorders (MSD) focus on work-related MSD. One group of interest is computer users, among whom several factors have been identified as being associated with MSD; physical/ergonomic factors, working technique, work organization, hours spent typing, psychosocial factors, and gender [7-9].

Studies among more general groups have indicated that work-related physical factors (heavy load, awkward positions, repetitive movements) and psychosocial factors (demands, control, mental stress), as well as several individual risk factors (age, gender, obesity, smoking, physical activity), are important in the understanding of neck/shoulder pain [2,3,10-14].

Some studies distinguish between specific and nonspecific MSD. Two recent studies indicated that certain physical risk factors were more strongly associated with specific disorders than with nonspecific pain in the upper limbs [12,13]. Another study, analyzing nonspecific regional MSD, identified psychosocial risk factors at work [1]. One recent study concluded that chronic and widespread musculoskeletal symptoms in neck or upper extremities were the main risk factors for self-reported generally reduced productivity due to musculoskeletal symptoms [15]. Several motivating reasons for further research are mentioned in the literature described above. More longitudinal and large-scale studies would permit conclusions about temporal relationships and are still requested in systematic reviews [5,16].

In the present study, three different regression models were used to analyze the longitudinal data, in order to address both the issue of better understanding of how risk factors affect musculoskeletal neck pain, and the issue of using relevant outcomes. Similar models have been proposed earlier, but either were not studied in combination or were not thoroughly discussed in terms of their benefits and their interpretation [14,17].

Musculoskeletal pain is not a clear event in time that happens at one time-point, f ex as death due to cancer or a first-time stroke. The pain comes and goes and this could be called recurrent pain [18]. The pain could also be long-lasting, even if the intensity sometimes varies, and this could be called persistent pain [18]. In the present study the course of pain could have either of these qualities. The term 'ongoing pain' is here defined as pain that was present during one year or part of that year, and then present also during the following year or part of it. That is, ongoing pain could be either recurrent or persistent pain, but which of these that are present can not be determined by the data in the present study. 'Developing pain' is here defined as when responders had no pain or only experienced pain periods lasting less than 8 days the year preceding baseline, and then at one-year follow-up had one or more periods of pain lasting for at least 8 days. Note that this is not necessary newly developed pain.

In this paper the theoretical frame-work will be based on the balance theory [19,20], in which health is supposed to be negatively affected from imbalance between various factors (at work and in leisure time). The demand-control-social support model [21] is a less extensive model, that here will be seen as included in a part of the balance theory. Research supports the relationships between psychosocial factors and musculoskeletal neck pain [5,16]. Note that here in a group of university students the lines between work and leisure time is not as clear as among other groups in working life.

Risks factors can have a short-term influence or a long-term one. For the purposes of the present study, short-term influence refers to the situation where the exposure and current pain are close together in time. Using different regression models and different outcome variables, the present paper tries to evaluate factors as short-respectively long-term.

The general aim of this paper was to analyze musculoskeletal pain in the neck or upper back, in a group of young adults. The explanatory variables considered were psychosocial factors, computer use, demands, and lifestyle. Specific aims were:

1. To determine which factors are long-term and short-term risk/protective factors for musculoskeletal pain.

2. To investigate whether the same factors are involved in the development of musculoskeletal pain as in ongoing musculoskeletal pain.

## Methods

### Material

The material in this study is based on a cohort, recruited in 2002, of university students enrolled in medical and IT-related studies. Baseline and yearly follow-up data were collected with an internet-based questionnaire; the baseline response rate was 70 percent. For a more extensive description of the study base see [22]. The present study uses data from the baseline, as well as the one-year and two-year follow-ups, among the 1204 respondents (628 women, 576 men) to the baseline questionnaire.

All subjects received written information concerning the study and their right to refuse to participate. Subjects agreed to participate by sending their approval by e-mail. This procedure was approved by the ethics committee of the Medical Faculty at the University of Gothenburg.

The outcome in this paper concerns pain in neck or upper back, according to the phrasing of the question in the questionnaire (Table 1). This is according to the consensus of the definition of the neck-region according to the Neck Pain Task Force [23]. We will for simplicity in the following text refer to neck pain in the meaning of our regions.

**Table 1:** Definition of the three outcome variables.

Outcome variable	Definition	Possible values
Pain at present	"Do you at present have pain in the upper part of your back/neck?" *Y = 1 if the participants answered yes to the question* *Y = 0 otherwise*	0, 1
A period of pain	"Have you, during the last year, had a period of pain lasting more than 7 days in the upper part of your back/neck?" *Y = 1 if the participants answered yes to the question* *Y = 0 otherwise*	0, 1
Number of years with pain	*Y = sum of 'a period of pain' over three years (baseline, one-year and two-year follow-ups)*	0, 1, 2, 3

Three outcome variables were used; pain at present, a period of pain, and the number of years with pain (Table 1).

For the outcomes 'pain at present' and 'a period of pain', there were no missing values at baseline; however, 77 respondents failed to answer either question at both follow-ups, and 242 answered the one-year follow-up but not the two-year follow-up. Hence, the one-year data included 1127 respondents and the two-year data included 885 respondents.

The explanatory variables used in this study were sorted into six blocks: background, lifestyle, demands, psychosocial factors, computer use and health. Some questions were combined into new variables; f ex high work/study demands (Table 2), due to multicollinearity problems.

**Table 2:** Explanatory variables sorted into six blocks.

	Original variables	New variables
Background	**Gender** *1 = women, 0 = men*	
	Length: *cm* Weight: *kg* Body mass index (BMI) = weight/length^2^	**Overweight**: *1 = BMI>25, 0 = BMI ≤ 25*
Lifestyle	"How many times PER WEEK do you have breakfast?" *0 time, 1 time, 2 times, 3 times, 4 times, 5 times, 6 times, 7 times*	**Breakfast regularly**: *1 = >5 days/week, 0 = ≤ 5 days/week*
	**Smoking**: "Have you been smoking daily or almost daily during the LAST 7 DAYS?" *1 = yes, 0 = no*	
	"Have you been using snuff daily or almost daily during the LAST 7 DAYS?" *yes, no*	**Snuff use**: *1 = uses snuff and does not smoke* *0 = does not use snuff, or uses snuff but also smokes*
	**Physical activity**: "Approximately how much time have you spent time on the following activity during the LAST 7 DAYS?: Exercise, training" *h/week*	
Demands	"What do you think of the demands of work/study performance in your work/study situation?" *Demands of work/studies are absolutely too high-Agree totally, Demands of work/studies are absolutely too high-Agree partially, Demands are neither to high nor to low, Demands of work/studies are absolutely too low-Agree partially, Demands of work/studies are absolutely too low-Agree totally* "Do the demands at work/studies affect your home and family life negatively?" *Very rarely, rarely, some times, often, very often*	**High work/study demands**: *0 = Demands of work/studies are not* *too high* *1 = Demands of work/studies are too high, but do not affect home life* *2 = Demands of work/studies are too high and affect home life*
	**High home life demands**: "Do the demands at home/from your family affect your work/studies negatively?" *Very rarely, rarely, some times, often, very often*	
	"Approximately how much time have you done the following activity during the LAST 7 DAYS?" Schoolwork (scheduled) Schoolwork (unscheduled) Gainful employment/paid work/practical training *h/week*	**Work/study time**: Sum of scheduled and unscheduled studies and paid work *h/week*
Psychosocial factors	"Take position to the following statement about your school/workplace: I get a long with superiors or teachers." *Exactly right, about right, not especially right, not right at all*	**Good superiors**: *1 = Exactly right, about right* *0 = Not especially right, not right at all*
	"Take position to the following statement about your school/workplace: I get a long with colleagues or fellow students." *Exactly right, about right, not especially right, not right at all*	**Good colleagues**: *1 = Exactly right, about right* *0 = Not especially right, not right at all*
Ergonomics Computer use pattern	"Do you use/have you been using the following equipment?" Ordinary desktop computer (PC) Laptop computer *Yes* Time using PC: *h/week*	Not included due to multicollinearity with No pause.
	"How many times during the LAST 7 DAYS have you been using the following equipment for more than 4 hours without a break (pause for more than 10 min)?" Personal computer *0 time, 1 time, 2–4 times, 5 or more times*	**Computer use pattern**: *0 = 0 time during last week* *1 = 1 time during last week* *2 = 2 or more times during last week*
Health	**Asthma**: "Have you received the diagnose asthma from a physician?" *1 = asthma diagnosed by physician, 0 = otherwise*	
	**Perceived stress**: "STRESS refers to a state when one feels tens, restless or worried or not being able to sleep at night because one thinks of problems all the time. Have you experienced this kind of stress for a period lasting longer than 7 days, during the LAST 12 MONTHS?" *1 = yes, 0 = no*	

Variables written in bold style are variables later used in the regression analysis.

The explanatory variable 'perceived stress' is based on the question validated by Elo [24].

### Statistical methods

For the outcomes 'pain at present' and 'a period of pain' raw baseline prevalence's were calculated. For the outcome 'number of years with pain' the proportion in each category was presented.

Three methods for regression analysis were used [14,25,26]: a marginal logistic regression model with an outcome of 'pain at present'; a Poisson regression model with an outcome of 'number of years with pain'; and a logistic Markov transitional model with an outcome of 'a period of pain'. The models are further explained below. In the first step of the analysis, each of the three models included one explanatory variable at a time together with gender (a simple regression model). In the second step of the analysis all those explanatory variables with P-values ≤ 0.2 were included in a multiple regression model. Gender was included as above. The choice of this limit, rather than P > 0.05, as an inclusion criteria for explanatory variables was made in order not to miss an explanatory variable with a possible association with musculoskeletal pain. We wanted to make sure to exclude only those variables that did not seem to be associated with musculoskeletal pain, or that were estimated with such uncertainty that no useful information was achieved. All analyses were performed using the PROC GENMOD procedure in the SAS statistical package (version 9.1, SAS Institute, Cary, NC, USA). Statistical significance was set at P ≤ 0.05. In all regression models, Wald type 3 P-values were used to assess the effect of each factor on the outcome variables. All P-values are two-sided.

For the binary outcomes 'pain at present' and 'a period of pain' odds ratios (OR) were achieved both from the simple regression models and from the multiple regression models. The OR and corresponding p-values derived from logistic regressions were used to test the association between different explanatory variables and the outcomes [27]. In addition proportion ratios (PR) were calculated. Here we use PR to denote the ratio between the proportions of those with the outcome, comparing the two exposure groups of interest. Other terminology in the literature for PR is among others; risk ratio (RR), probability ratio (PR), prevalence proportion ratio (PPR) [28-30]. The proportion ratios were calculated in order to estimate the magnitude of the exposure effect. The calculations were based on the parameter estimates in the simple logistic regression (as suggested in [27,31]). These calculations produces one estimate of PR for each possible combination of other explanatory variables included in the regression, and were therefore not calculated in the case of the multiple regression models. Here we calculated one PR for women and one PR for men and the adjusted PR is then the mean of these two PR's [25]. There is no straightforward procedure known to the author for providing appropriate CI for the indirect PR and it is out of the scope of this paper. Therefore in the results section estimates of OR, 95% CI and their p-values will be presented in combination with the estimates of the gender adjusted PR.

### Marginal model for binary outcome (short-term effect)

The binary outcome 'pain at present' was modelled with a marginal logistic model [32]. The aim was to investigate whether the risk factors had a short-term effect (outcome and exposure occurring close together in time). A marginal model takes into account the repeated measurement structure of the data by modelling the correlation structure; it is an appropriate choice of model when focusing on population averages [32]. For each person, three observations of 'pain at present' were included in the analysis, one for each year. The explanatory variables were chosen from the same year as the outcome, as the intent was to determine short-term risk factors. If the explanatory variable varied over the three years, and the outcome also varied with the same pattern, the results were interpreted as an indication of a short-term effect. If the explanatory variable varied over the three years, and the outcome variable also varied but with a different pattern, then no association could be found and this was interpreted as a lack of short-term effect. If both the explanatory variable and the outcome were stable over time then no discrimination could be made between short-term or long-term effects.

The response variable Y (here 'pain at present') was assumed to follow a Bernoulli distribution with parameter p, where p = *P*(*Y *= 1) and



where *x*_1_, *x*_2_, ..., *x*_*k *_are the explanatory variables, index i refers to the individual, and index t refers to the time point (0, 1, 2). The odds ratio for the effect of x_j _is exp(βj). The quasi-likelihood equations, which are known as generalized estimating equations (GEE), are used to take into account the repeated structure. The SAS procedure PROC GENMOD (link = logit, distribution = binomial, working correlation structure = exchangeable) was used. The GEE method gives consistent parameter estimates even if the correlation structure is misspecified; see [32] p. 468). However, it should be noted that the empirically-based standard errors which are used when making inferences about parameters with Wald statistics and asymptotic normality of estimators tend to underestimate the true errors unless the sample size is quite large [32] p. 467-468). Analysis was performed with both the exchangeable and the unstructured working correlation structure. The unstructured working correlation structure, which is more flexible then the exchangeable, was chosen as it gave slightly smaller P-values.

### Poisson model for counts (long-term effect)

A Poisson model was used to analyze 'number of years with pain'. Thus, the longitudinal data has been summarized into one value for each person. For the explanatory variables the baseline values were used. The explanatory variable then precedes all the yearly outcomes. The purpose of the analysis was to identify possible long-term risk factors.

The observations, Y (here 'number of years with pain'), were assumed to follow a Poisson distribution with expected value μ, where



where *x*_1_, *x*_2_, ..., *x*_*k *_are explanatory variables and index i refers to the individual.

A model allowing for over-dispersion was used; that is, a Poisson distribution where the variance was larger than the expected value. The PROC GENMOD procedure of SAS was used (link = Poisson, distribution = Poisson, scale = Pearson). The analysis indicated some over-dispersion (over-dispersion parameter = 1.1), and by allowing this over-dispersion, the standard errors were not too small.

The response variable is based on the sum of the binary outcome for the three years. The sum of binary variables follows a Poisson distribution if the binary variables are independent, but here, in fact, the outcomes from one person are likely to be correlated. Hence, the true effect of exposure might be underestimated (as was observed in [33], and this must be remembered when interpreting the results.

### Markov transitional model for binary outcome (developing and ongoing pain)

The binary outcome 'a period of pain' was modelled with a Markov transition model. The aim of this analysis was to identify those factors that might have a long-term influence, by investigating whether an exposure at baseline was associated with the development or recurrence of pain during the following year. The response variable 'a period of pain' was assumed to follow a Bernoulli distribution, with parameter p (p = P(*Y*_*it *_= 1)). First, the development of pain was studied, by only considering those who were pain-free at baseline (Y_0 _= 0) and modelling the probability of having pain at follow-up:



where *x*_j, t _refers to explanatory variable j at time t.

Next, the recurrence of pain was studied by considering those who had pain at baseline (Y_0 _= 1), and modelling the probability of having pain at follow-up:



## Results

### Descriptive

There were some differences between men and women in the distribution of the explanatory variables (Table 3). All analyses were therefore adjusted for gender (men, women).

**Table 3:** Descriptive statistics for the total group of individuals.

	Baseline values		Intra-individual variability
	Mean (min; max)		Average individual sd
	Women	Men	**(**sd of average sd)
Work/study time (h/week)	36 (0; 82)	35 (0; 98)	11.6 (8.06)
Physical activity (h/week)	4 (0; 40)	4 (0; 40)	2.0 (2.32)

	%	%	Proportion of persons in same category all 3 years, %
	
Overweight	8	15	90
Breakfast regularly	85	79	83
Snuff use, non-smoking	2	10	96
Smoking	6	5	94
High work/study demands			43
*No too high demands*	49	71	
*High demands, not affecting home life*	34	20	
*High demands, affecting home life*	17	9	
High home life demands	3	6	88
Good superiors	95	93	87
Good colleagues	94	93	88
Computer use pattern			49
*No 4 h periods without a break (0)*	66	48	
*One 4 h period without a break (1)*	12	15	
*At least two 4 h periods without a break (2)*	22	37	
Asthma	8	7	96
Perceived stress	65	46	52

Baseline values and variability within individuals over the years. Number of women at baseline: 579–586*, number of men at baseline: 538–540. The total number of respondents varied because of incomplete data.

The variables which differed over the three years were work/study time, physical activity (to some extent), high work/study demands, computer use pattern and perceived stress; it was thus possible to test these variables for short-term effect on musculoskeletal pain (Table 3).

The prevalence of 'a period of pain' at baseline, 23 percent, seems to be in the same range as the prevalence of upper back and neck pain after work among the younger section of the Swedish workforce (16–29 years) in 2003 [34]. The prevalence of pain at present was just slightly lower, 20 percent. Among the respondents, 15 percent developed pain between baseline and the one-year follow-up, and 52 percent had ongoing pain. If looking over the whole three year period; a little more then half of the respondents, 61 percent, did not report any year with a period of pain, while 20 percent reported one year with pain period, 11 percent reported two years with pain period, and 8 percent reported pain period all three years.

### Regression analysis

The first step in the regression analyses was to include one explanatory variable at the time together with gender (results presented under OR and PR or RR in Table 4, 5, 6). The second step was a multiple regression where gender, together with explanatory variables with p ≤ 0.2 in step 1, was included.

**Table 4:** Marginalmodels – analyses of pain at present.

**Explanatory variables from baseline**	**Exposed cases**	**Simple model**		**Adjusted model**			**Proportion ratio**
		**Odds ratio**	**P-value**	**Odds ratio**	**P-value**	**95% CI**	
Women/men	160	2.9	< 0.001	2.6	< 0.001	2.02; 3.41	2.4
Overweight	22	1.1	0.463				1.1
Breakfast regularly	168	0.64	0.001	0.77	0.058	0.593; 0.995	0.71
Snuff use	5	-	-				-
Smoking	23	2.2	< 0.001	2.0	0.003	1.35; 3.00	1.8
Physical activity		0.98	0.157	0.99	0.318	0.967; 1.01	0.99
High work/study demands (ref: not too high)	104		< 0.001		0.004		
*Not affecting home* *life*	71	1.4		1.3		1.06; 1.61	1.3
*Affecting home life*	45	1.8		1.5		1.16; 1.99	1.6
High home life demands	12	1.5	0.020	1.2	0.243	0.868; 1.77	1.4
Work/study time		1.0	0.266				1.0
Good relationship with superiors	206	0.78	0.139	0.89	0.507	0.624; 1.26	0.82
Good relationship with colleagues	207	0.76	0.175	0.92	0.700	0.614; 1.39	0.81
Computer use pattern (ref: 0)	120		< 0.001		0.012		
*One 4 h period without a break*	24	1.0		1.0		0.748; 1.34	1.0
*At least two 4 h periods without a break*	76	1.6		1.4		1.11; 1.71	1.4
Asthma	23	1.2	0.360				1.1
Perceived stress	163	1.8	< 0.001	1.6	< 0.001	1.34; 2.01	1.6

All odds ratios are adjusted for gender (using men as the reference category). For the adjusted odds ratios both p-values and 95% confidence intervals (CI) are presented. The total number of respondents varied between N = 737 – 885, because of incomplete data. Estimates were not calculated for explanatory variables with five or fewer exposed cases.

**Table 5:** Poisson models – analyses of number of years with pain.

**Explanatory variables from baseline**	**Exposed cases**	**Simple model**		**Adjusted model**		
	**(Y>0)**	**Rate ratio**	**P-value**	**Rate ratio**	**P-value**	**95% CI**
Women/men	245	1.7	< 0.001	1.6	< 0.001	1.37; 1.79
Overweight	36	0.95	0.525			
Breakfast regularly	280	0.83	0.015	0.96	0.612	0.809; 1.13
Snuff use	9	0.84	0.126	1.0	0.824	0.856; 1.21
Smoking	23	1.4	0.045	1.2	0.210	0.862; 1.78
Physical activity		0.99	0.158	1.0	0.926	0.988; 1.01
High work/study demands (ref: not too high)	189		0.004		0.047	
*Not affecting home life*	92	1.0		0.94		0.822; 1.08
*Affecting home life*	67	1.4		1.3		0.998, 1.58
High home life demands	21	1.4	0.039	1.1	0.532	0.802; 1.51
Work/study time		1.00	0.746			
Good relationship with superiors	327	0.80	0.099	0.87	0.321	0.652; 1.17
Good relationship with colleagues	322	0.88	0.284			
Computer use pattern (ref: 0)	185		0.003		0.024	
*One 4 h period without a break*	53	1.2		1.1		0.953; 1.36
*At least two 4 h periods without a break*	110	1.2		1.2		1.04; 1.37
Asthma	36	1.4	0.006	1.3	0.102	0.934; 1.69
Perceived stress	239	1.4	< 0.001	1.3	< 0.001	1.17; 1.51

All rate ratios are adjusted for gender (using men as the reference category). For the adjusted odds ratios both p-values and 95% confidence intervals (CI) are presented. The total number of respondents varied between N = 677 – 801, because of incomplete data.

**Table 6:** Developing pain: Markov transition models – analyses of a period of pain at one-year follow-up, for those without a period of pain at baseline.

**Explanatory variables from baseline**	**Exposed cases**	**Simple model**		**Adjusted model**			**Proportion ratio**
		**Odds ratio**	**P-value**	**Odds ratio**	**P-value**	**95% CI**	
Women/men	86	3.0	< 0.001	3.1	< 0.0001	2.00; 4.82	2.5
Overweight	10	0.80	0.522				0.82
Breakfast regularly	103	0.81	0.423				0.84
Snuff use	5	-	**-**				-
Smoking	7	1.2	0.685				1.1
Physical activity		0.99	0.599				0.99
High work/study demands (ref: not too high)	62		0.126		0.296		
*Not affecting home life*	48	1.4		1.4		0.913; 2.20	1.4
*Affecting home life*	16	1.5		1.1		0.596; 2.12	1.3
High home life demands	8	2.2	0.063	2.2	0.080	0.912; 5.07	1.9
Work/study time		1.0	0.889				1.0
Good relationship with superiors	116	0.67	0.280				0.72
Good relationship with colleagues	114	0.56	0.098	0.72	0.373	0.354; 1.48	0.62
Computer use pattern (ref: 0)	64		0.016		0.021		
*One 4 h period without a break*	20	1.6		1.7		0.941; 2.94	1.5
*At least two 4 h periods without a break*	42	1.9		1.8		1.16; 2.89	1.7
Asthma	13	2.0	0.046	2.0	0.052	0.996; 3.91	1.7
Perceived stress	84	1.9	0.002	1.7	0.011	1.13; 2.63	1.7

All odds ratios are adjusted for gender (using men as the reference category). For the adjusted odds ratios both p-values and 95% confidence intervals (CI) are presented. The total number of respondents varied between N = 267 – 326, because of incomplete data. Estimates were not calculated for explanatory variables with five or fewer exposed cases.

In the multiple regression model for 'pain at present' gender (women compared to men) was a statistically significant explanatory variable for neck pain (Table 4). The risk factors were smoking, high work/study demands, computer use pattern and perceived stress; high home life demands was a risk factor in the simple model, but in the multiple regression turned out not statistically significant (Table 4). Breakfast regularly, as a protective factor, was only close to statistically significant in the multiple model, though statistically significant in the simple model of pain at present (Table 4).

In the multiple regression model for 'number of years with pain' the statistically significant explanatory variables were gender (women compared to men), computer use pattern, high work/study demands and perceived stress (Table 5). In the simple models smoking, high home life demands and asthma were risk factors and breakfast regularly was a health factor, but none of these were statistically significant in the multiple regression model (Table 5). It should be noted that the rate ratios from the Poisson model for 'number of years with pain' are interpreted as the ratios of expected numbers of years with pain, and are not comparable in magnitude with the corresponding odds ratios.

In the multiple model for developing musculoskeletal pain during the last year ('a period of pain') the statistically significant explanatory variables were gender (women compared to men), computer use pattern and perceived stress (Table 6). Asthma also was a risk factor for developing pain in the simple model, but only close to statistically significant in the multiple model (Table 6).

In the multiple model for ongoing musculoskeletal pain during the last year ('a period with pain') none of the factors were statistically significant, but perceived stress was close (Table 7). In the simple model the explanatory variables women (compared to men) and perceived stress were statistically significant (Table 7).

**Table 7:** Ongoing pain: Markov transition models – analyses of a period of pain at one-year follow-up, for those with a period of pain at baseline.

**Explanatory variables from baseline**	**Exposed cases**	**Simple model**		**Adjusted model**			**Proportion ratio**
		**Odds ratio**	**P-value**	**Odds ratio**	**P-value**	**95% CI**	
Women/men	106	1.9	0.027	1.6	0.157	0.837; 3.01	1.4
Overweight	14	0.87	0.848				0.96
Breakfast regularly	114	1.5	0.202				1.2
Snuff use	4	-	-				-
Smoking	12	1.4	0.231				1.1
Physical activity		0.98	0.684				0.99
High work/study demands (ref: not too high)	64		0.606				
*Not affecting home life*	40	1.2					1.1
*Affecting home life*	32	1.3					1.2
High home life demands	5	-	-				
Work/study time		0.98	0.095	0.98	0.093	0.962; 1.00	0.99
Good relationship with superiors	126	0.49	0.206				0.73
Good relationship with colleagues	129	1.4	0.545				1.2
Computer use pattern (ref: 0)	81		0.568				
*One 4 h period without a break*	18	0.98					0.99
*At least two 4 h periods without a break*	37	0.75					0.86
Asthma	17	1.8	0.198	1.9	0.205	0.708; 5.00	1.3
Perceived stress	108	1.9	0.029	1.8	0.064	0.965; 3.50	1.4

All odds ratios are adjusted for gender (using men as the reference category). For the adjusted odds ratios both p-values and 95% confidence intervals (CI) are presented. The total number of respondents varied between N = 214–264, because of incomplete data. Estimates were not calculated for explanatory variables with fewer than five exposed cases.

From the simple regression models we have that the prevalence for neck pain among women were 28 percent (pain at present), 22 percent (developing pain) and 56 percent (ongoing pain); and the prevalence for neck pain among men were 12 percent (pain at present), 9 percent (developing pain) and 41 percent (ongoing pain).

## Discussion

### Principal findings

Pain was more prevalent among women than men for all outcome measurements, except for ongoing pain where result were indistinct. Perceived stress was a risk factor regarding developing pain, and was both a short-term and a long-term risk factor. Moreover, the results showed that high work/study demands were a short-term and long-term risk factor for neck pain. Computer use pattern was a risk factor for developing pain, but also both a short- and a long-term risk factor. The above findings, regarding type of factor and direction of association, are consistent with a systematic review concerning neck pain [5] and with results in more recent studies [3,8,11,35]. Smoking was a risk factor for pain at present. Less certain results regarding possible risk factors were that smoking was, in the simple model, associated with number of years with pain. High home life demands were, in the simple models, associated with pain at present and with number of years with pain. Asthma was, in the simple models, associated with developing pain and number of years with pain. In an earlier study, association between asthma and low back pain was shown [36].

Results for ongoing pain were more uncertain, possibly due to the lower number of observations as only those reporting a period of pain in the baseline questionnaire were included. For ongoing pain perceived stress was a risk factor in the simple model, but only close to statistical significance in the multiple model. In the simple model gender was associated with ongoing pain, but in the multiple model the result was inconclusive. Note though that for all explanatory variables in the multiple regression the estimated OR:s were of the same size as in the simple regressions. The only exception was for gender, where the OR for women compared to men decreased slightly in the multiple regression compared to the simple regression.

In all analyses, work/study time and physical activity (measured as hours per week) seemed to have very small or no effect (0.98 ≤ OR ≤ 1.0, 0.99 ≤ PR ≤ 1.0, 0.99 ≤ RR ≤ 1.0) and were not statistically significant in any analyses. This was also the case for overweight (0.87 ≤ OR ≤ 1.0, 0.82 ≤ PR ≤ 1.1, RR = 0.95).

The proportion of women developing pain was more than twice the proportion of men who develop pain (PR = 2.5). For ongoing pain, the corresponding proportion ratio was 1.4, meaning that the proportion neck pain for women were in this case much closer to the proportion for men. This is consistent with recent systematic reviews that reported that women in the general working population were slightly more likely to report neck pain compared with men, but that the evidence is inconsistent regarding the role of gender in recovery from neck pain [16,37]. It would be interesting to investigate, in further studies, whether the differences between women and men in musculoskeletal pain mainly occur in the development of pain. A hypothesis worth testing would be that there is still some unexplained difference between men and women in the development of pain, while differences between men and women who already have pain can to a larger extent be explained by exposure to known risk factors. Suggested reasons in earlier studies behind the gender differences in MSD include the over-representation of women in sedentary, and repetitive work, and the persistent gender imbalance in domestic work [38]. As the present study group consisted of young university students mainly without children the above explanation does not seem to apply here.

### Strengths and weaknesses of the study

One of the limitations of the present study is that the outcome variables do not incorporate the disability or burden of the pain. This study should be seen as a first step towards stronger analysis of musculoskeletal outcome by using longitudinal data.

One of the aims of this study was to classify factors as having long-term or short-term effects. In order to allow interpretation of the explanatory variables as long-term or short-term effects, we investigated the stability of these variables over time. High work demands, computer use pattern, and perceived stress varied over the years, and could therefore be investigated regarding short-term effects in the model for pain at present and regarding long-term effects in the model for number of years with pain, the model for development of pain, and the model for ongoing pain. Factors that were not changeable (gender), or that could vary but did not in the present material (breakfast regularly, smoking) could not be investigated concerning short-term effects.

The type of self-reported neck pain outcome used in the present study is noted to be sensitive to effects of seasonal variation at different follow-ups [39]. In the present study the time of the year for the data collection was the same to avoid this seasonal effect.

As in many studies, both the outcome and the exposure were self-reported. This could result in recall bias; that is a tendency for individuals who have a high level of pain to over report their exposure, and the reverse tendency among the pain free individuals. Interpretation of effects in the analysis of the marginal model should be made with caution, as the outcome and exposure were measured at the same time. However, as the questions about exposure referred to either the previous week or to an undefined time period previous to the measurement, it is possible to interpret the results as indications of temporal effect.

Due to the use of different regression models and different outcomes, the integrated conclusions drawn from these analyses are more informative than the conclusions from a single one of these regressions. The different outcome variables used here represent temporary or ongoing musculoskeletal pain.

### Meaning of the study: possible implications

The most consistent risk factors were perceived stress, high work/study demands and computer use pattern. In the frame work of the balance theory this could be interpreted as factors measuring some aspects of lack of balance between load and recourses or possibility for recovery. Computer use pattern to some degree measures the constant low intensity physical exposure of computer work without breaks for recovery. High work/study demands when negatively affecting home life could represent demands too high to allow for sufficient recovery after work. Perceived stress could be interpreted as the persons own perception of imbalance between loads and resources. This interpretation of the most important risk factors suggests that the preventive as well as the curative work, related to musculoskeletal neck pain, should focus on the balance between load and resources/recovery.

### Future research

These results should be confirmed in further longitudinal studies, where it would be desirable to investigate an outcome that includes the dimension of disability or burden and also to perform intervention studies to better understand causal effects.

## Conclusion

Perceived stress, high work/study demands and computer use pattern (break pattern) were short-term risk factors as well as long-term risk factors for musculoskeletal neck pain. Perceived stress and computer use pattern were involved in developing neck pain. By the use of different regression models the different aspects of the neck pain pattern could be addressed and the risk factors impact on the pain pattern was identified.

## Competing interests

The authors declare that they have no competing interests.

## Authors' contributions

AGE: Designing the study's analytic strategy and analyzes, and preparation of the main body of the text. EMA: Supervised the statistical methodology and participated in preparation of the text. MH: Designing the study, responsible for the cohort, and supervised the applied parts of the study and discussion over results. All authors read and approved the final manuscript.

## Pre-publication history

The pre-publication history for this paper can be accessed here:


